# Loss associated with subtractive health service change: The case of specialist cancer centralization in England

**DOI:** 10.1177/13558196221082585

**Published:** 2022-04-26

**Authors:** Georgia B Black, Victoria J Wood, Angus I G Ramsay, Cecilia Vindrola-Padros, Catherine Perry, Caroline S Clarke, Claire Levermore, Kathy Pritchard-Jones, Axel Bex, Maxine G B Tran, David C Shackley, John Hines, Muntzer M Mughal, Naomi J Fulop

**Affiliations:** 1 Principal Research Fellow, Department of Applied Health Research, 4919 University College London, London, UK; 2 Research Associate, Department of Applied Health Research, 4919 University College London, London, UK; 3 Senior Research Fellow, Department of Applied Health Research, 4919 University College London, London, UK; 4 Senior Research Fellow, Department of Targeted Intervention, University College London, London, UK; 5 Research Fellow, Applied Research Collaboration Greater Manchester/Division of Population Health, Health Services Research and Primary Care, 5292 University of Manchester, Manchester, UK; 6 Senior Research Fellow, Research Department of Primary Care & Population Health, University College London, London, UK; 7 Executive Director of Operations, North Central London Cancer Alliance, 8964 University College London Hospitals NHS Foundation Trust, London, UK; 8 Professor of Paediatric Oncology, North Central London Cancer Alliance, University College London Hospitals NHS Foundation Trust, & University College London Partners, London, UK; 9 Department of Urology, 4965 Royal Free London NHS Foundation Trust London, London, UK; 10 Consultant Clinical Lead Specialist Centre for Kidney Cancer, Division of Surgery and Interventional Science, University College London, London, UK; 11 Senior Lecturer in Renal Cancer Surgery, Division of Surgery and Interventional Science, University College London, London, UK; 12 Specialist Centre for Kidney Cancer, Royal Free London NHS Foundation Trust, London, UK; 13 Director & Medical Lead, Greater Manchester Cancer; Clinical Lead Manchester Academic Health Science Centre, 5292 University of Manchester, Manchester, UK; 14 Consultant Urological Surgeon and Urology Pathway Director, Division of Surgery and Interventional Science, University College London, London, UK; 15 Honorary Clinical Professor, Division of Surgery and Interventional Science, University College London, London, UK; 16 Professor of Health Care Organisation and Management, Department of Applied Health Research, 4919 University College London, London, UK

**Keywords:** Major system change, centralization, leadership, organizational loss

## Abstract

**Objective:**

Major system change can be stressful for staff involved and can result in ‘subtractive change’ – that is, when a part of the work environment is removed or ceases to exist. Little is known about the response to loss of activity resulting from such changes. Our aim was to understand perceptions of loss in response to centralization of cancer services in England, where 12 sites offering specialist surgery were reduced to four, and to understand the impact of leadership and management on enabling or hampering coping strategies associated with that loss.

**Methods:**

We analysed 115 interviews with clinical, nursing and managerial staff from oesophago-gastric, prostate/bladder and renal cancer services in London and West Essex. In addition, we used 134 hours of observational data and analysis from over 100 documents to contextualize and to interpret the interview data. We performed a thematic analysis drawing on stress-coping theory and organizational change.

**Results:**

Staff perceived that, during centralization, sites were devalued as the sites lost surgical activity, skills and experienced teams. Staff members believed that there were long-term implications for this loss, such as in retaining high-calibre staff, attracting trainees and maintaining autonomy. Emotional repercussions for staff included perceived loss of status and motivation. To mitigate these losses, leaders in the centralization process put in place some instrumental measures, such as joint contracting, surgical skill development opportunities and trainee rotation. However, these measures were undermined by patchy implementation and negative impacts on some individuals (e.g. increased workload or travel time). Relatively little emotional support was perceived to be offered. Leaders sometimes characterized adverse emotional reactions to the centralization as resistance, to be overcome through persuasion and appeals to the success of the new system.

**Conclusions:**

Large-scale reorganizations are likely to provoke a high degree of emotion and perceptions of loss. Resources to foster coping and resilience should be made available to all organizations within the system as they go through major change.

## Introduction

Centralization of specialist services moves clinical or surgical activity to high volume centres, with subtractive change (loss of activity) from others, and is an example of ‘major system change’ (MSC): a system-level intervention coordinated by multiple organizations and care providers.^1,2^ Centralization is often implemented with the aim of improving quality of care and outcomes through increased volume in a smaller number of units, while addressing challenges of workforce capacity and costs.^1^ Research on this topic has identified aspects of MSC, such as factors associated with successful implementation,^3^ the reasons why it works better in some areas than others^2^ and how different approaches to MSC influence the outcome.^1^ There is also a growing body of research challenging the underlying assumptions of MSC, and examining its social, cultural and political aspects.^4,5^

Studies in the health care sector have shown there are emotional costs associated with organizational change. For example, Fulop et al. found that mergers can cause stress due to uncertainty and change, increased workload and perceptions of being ‘taken over’.^6^ Other negative emotions and actions that stem from organizational change include change fatigue,^7^ bullying^8^ and feelings of loss or grief, even when clinical outcomes are improved by the change.^6,9,10^ Feelings such as insecurity and anxiety may stem from a loss of leadership or fear of not being able to meet new requirements of a role.^10^ Emotions associated with organizational change may be experienced differently depending on a person’s position within an organization.^11^ Feelings of loss may be driven by employee identity, which is disrupted when their organization changes or a connection to it is lost,^12^ resulting in stress.^13^

Change can provoke particularly strong emotional reactions when a part of the work activity or environment is lost.^14^ Negative emotions are exacerbated by certain subtractive change processes, such as the threat of redundancy, restricted involvement in decision-making or consultation, lack of support and changes in job roles that increase workload or work complexity.^13,15,16^ These emotions can be attenuated over time, especially if the new organization is deemed to be preferable. Leaders and managers can help mitigate negative emotions in relation to subtractive change by offering support.^17^ House presents four different types of support: instrumental (material assistance in response to specific needs), informational (advice or guidance), emotional (offering psychological support) and appraisal (offering understanding and validation).^17^ However, leaders can be unprepared and untrained in providing support^6^ or worse and can stigmatize those that show stress or emotion.^18^ Conversely, leaders who become adept at responding to emotional reactions within the system contribute to its robustness and resilience to change.^13^

There is evidence that subtractive change can lead to staff leaving an organization, which, in turn, can threaten the sustainability of expertise within the organization’s workforce and, indeed, the very size of its workforce.^19^ There is little evidence on how these issues affect whole teams, how leaders mitigate and manage them and/or how leaders attempt to reduce threats to implementation.

### MSC in specialist cancer surgery in London

Following international and national drives to concentrate specialist cancer surgery in high volume centres (e.g. Gooiker et al., 2011),^20^ there was a national policy drive to reorganize services in England.^21,22^ In 2010, strategic decision-makers in London and Manchester embarked on this centralization process for a number of specialist cancer services, including bladder, prostate, renal, oesophago-gastric, head and neck, haematological and brain cancers.^23,24^ The proposed benefits were increased quality of care through enhanced specialist expertise, standardized diagnostic pathways and treatment options, reduced postoperative mortality and variation in care and an integrated network of training and research opportunities across a network of hospitals.^19^ As a result, patients would be provided with better access to ‘world-leading’ services for their surgery and care.^23^

From 2012 to 2016, changes to the provision of cancer services in South of England were planned and implemented.^25^ In a previous paper, we described the nature of the changes.^25^ Four hospital sites were required to develop as specialist centres and 12 had to cease providing specialist surgical activity (see Online Supplement 1, Table S1). The latter were designated as local centres, providing diagnostics and follow-up services (see Online Supplement 1, Table S2).

As part of these changes, an organizational network was created to both lead the implementation of the changes and govern the system once implemented.^19^ The changes had an overarching programme governance, bringing together independent leadership by a central organization,^26^ and ‘frontline’ leadership by clinicians from the various Trusts that run the hospitals, organized by cancer site.^19^

This paper is drawn from a wider evaluation of the centralization programme, [RESPECT-21], which analyses planning, implementation and sustainability and the impact of centralization on clinical processes, clinical outcomes, costs and cost-effectiveness and patient experience.^25^ Our research focussed on renal, prostate, bladder and oesophago-gastric services. Our aim was to understand experiences of loss associated with MSC and to understand the impact of leadership and management on enabling or hampering coping strategies associated with that loss.

## Methods

This is a qualitative study drawing on interviews with key stakeholders in the centralization process, non-participant observations and document review. The study was primarily focussed on interviews drawn from sites that lost surgical activity, but also included interviews with members of the central leadership team. Our research team included health services researchers as well as clinicians from the services being studied. This facilitated recruitment of interviewees and provided insights into our findings. While clinical authors were consulted about the results and contextual factors, the non-clinical authors maintained a critical distance and maintained independence in their interpretations of the data.

### Data collection

The interviews were semi-structured, conducted by three experienced qualitative researchers [CV, VJW, GBB]. A topic guide was developed by the research team to guide the interviews, based on knowledge about the processes of centralization from previous work undertaken by some of the researchers on the project. Further details on our data collection and recruitment procedures are detailed in a prior publication from this study.^19^ Topics covered the various stages of the changes, including the proposal to change, planning and implementation.

We analysed 115 interviews with 81 participants (some participants took part in follow-up interviews), conducted between 2016 and 2019. The profile of the interviewees is given in Table 1. The interview data came from a wider dataset, collected as part of our larger evaluation work. All interviews were conducted by the authors in person or via telephone, depending on the preference of the participant. All interviews were digitally recorded and then professionally transcribed verbatim. Our interviews gave retrospective information, in that they were undertaken after the implementation of the centralization programme had been completed.

**Table 1.** Profile of interviewees.

Interviewee group	Number
Network managers and other network staff members	8
Local context^a^	9
Patient representatives	3
Urology Pathway Board^b^ members	4
Oesophago-gastric (OG) Pathway Board^b^ members	4
OG clinicians^c^ from hospital organizations (specialist and local centres)	14
Urology clinicians^c^ from hospital organizations (specialist and local centres)	30
OG managers from hospital organizations (specialist and local centres)	2
Urology managers from hospital organizations (specialist and local centres)	7
Total	81

^a^Includes commissioners (staff involved in the planning and purchase of NHS and publicly funded social care services), academics, staff members from organizations outside of the network and representatives from patient groups.

^b^Pathway Boards define best practice along the patient pathway and aim to facilitate delivery of these across the network. These boards are led by clinical pathway directors and include representation from patients, primary care and cancer professionals from all NHS hospitals in the network/system.

^c^Clinicians includes surgeons, nurses, oncologists, allied health professionals, pathologists and radiologists. No clinical trainees or students were included.

We were supplied with documentary evidence (over 100 documents) by people involved in the planning and implementation of the centralization and by our own online searching. We conducted non-participant observation (134 hours) of relevant board meetings, specialist multidisciplinary team meetings at specialist and local centres and other events associated with the centralization.

### Sample

We used fieldwork and snowball sampling to create a purposive sample of key informants involved in the centralization process.^27^ Informants were chosen to obtain perspectives of those planning and leading change and those delivering specialist cancer surgery, to understand the strategies employed and the perceived impact on delivery of care.

### Data analysis

During initial analysis by three authors [GBB, VJW, CV], all transcripts, field notes from observation and documents were thematically analysed, identifying a recurring theme of perceptions of loss.^28^ Following this, five authors subsequently reanalysed and organized the data into a framework reflecting the different types of loss experienced [GBB, VJW, AIGR, CV, NJF].^29^ As part of this process, the analysis was guided by literature conceptualizing organizational change and loss as a stressor by the first authors [GBB, VJWGBB, VJW],^10,13,17,30^ and drawing on psychological stress-coping theory.^31^ All authors contributed to interpretation and contextualization of the data.

### Ethical approval

The study [Reference 15/YH/0359] received ethical approval in July 2015 from the Proportionate Review Sub-committee of the NRES Committee Yorkshire & the Humber-Leeds (Reference 15/YH/0359).

## Results

**Figure 1.**
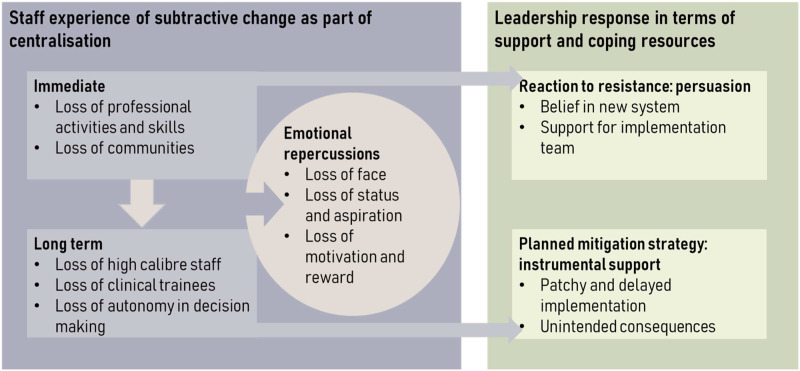
Relationships between subtractive change, emotional repercussions and support offered.

Overall, our findings suggest that feelings of loss were time-dependent, with subtractive changes being anticipated during the planning and experienced during the implementation phases of the centralization (see Figure 1). For this reason, our interview quotations are presented with the month and year of the interview.

In the long term, staff at local sites perceived their organizations as being less attractive than before centralization. This was demonstrated by the organizations’ difficulty retaining or attracting staff and trainees (see Figure 1). All subtractive changes provoked emotional ‘chain reactions’ in both the short and long term, with staff experiencing personal loss of status, autonomy and motivation.

Figure 1 also demonstrates that the central leadership of the centralization offered support, but mostly in the form of instrumental help, rather than emotional support. Emotional support was focussed on the staff members leading the implementation, rather than those experiencing subtractive change. There was some evidence of using interventions to try to suppress resistance.

### Immediate subtractive change: Loss of activity, skill and continuity

#### Loss of professional activity and skill

Local units broadly ceased to provide specialist surgical activity following the centralization. Being able to practise specialist procedures was seen as a particularly important component of surgeons’ roles. For individuals ceasing specialist surgeries, the long-term impact included loss of skills:

I noticed it when I came to [Trust]…the de-skilling of the local surgeons…If one loses the procedures, it’s more inconvenient for the patient, but also it has potential impact on the finances of the hospital and de-skilling of local surgeons. (surgeon, May 2017)

Even for surgeons continuing to practise some surgery at the specialist sites, other skills were lost, such as postoperative care, counselling and support. This surgeon was carrying out specialist surgery at a Trust that was geographically far from their main employer, meaning that they could not visit patients on ward rounds:

I wouldn’t have anything on my job plan to say that I was going to do ward rounds, before or after the surgery. And it would just be to actually go and do the surgery, like any technician. And it just felt very difficult because I wouldn’t know whose patients I would be operating on. I felt I wouldn’t be able to counsel my own patients, and then wouldn’t be able to tell them that I was definitely going to be doing their surgery…I just felt like I didn’t really want to be part of a service that I just felt I wasn’t able to control the circumstances of my work really. (surgeon, October 2017)

Indeed, despite opportunities to continue specialist surgeries, the centralization still provoked feelings of loss for surgeons, due to the changes in postoperative teamwork. Therefore, some chose to step down, moving to private practice or giving up surgery.

#### Loss of communities

One document supporting centralization we sighted said the process was designed for local and specialist sites to be ‘integrated’ and ‘coordinated’, with ‘‘Hub’ and ‘spoke’ surgeons to work together as a team’ (service specification document for oesophago-gastric cancer). But specification documents included few instructions for achieving this beyond videoconferencing, handovers and joint multidisciplinary team meetings.

Unsurprisingly, then, participants saw their local clinical environments as communities that had been destabilized by the centralization, resulting in reduced workplace interactivity and continuity of care. Maintaining close continuous contact with patients and families throughout their care was seen to be an important part of the community structure:

It was nice because I would visit them daily. Because you get to know the patient, you get to meet their families as well and you support the family…that aspect of it I miss greatly. (clinical nurse specialist, August 2018)

The same interviewee said that, after the reorganization, these aspects had been lost and that the reorganization added greater distance to the relationship between patients and specialist nurses working in the local hospitals:

I think what’s lost in all this is…the rapport the cancer-vulnerable patients develop with their professionals in the local hospital. (clinical nurse specialist, February 2017)

Surgeons also valued the rapport built up through sustained contact with patients. Even surgeons who supported the concept of centralization felt regret at the loss of continuity of care, and this persisted two to three years into the reconfiguration, as revealed in our follow-up interviews. Instead, patients were now attended by a network of consultants across different Trusts. For example, one surgeon in a local hospital explained that they would currently assess a patient, who would then be seen by a different consultant in the multidisciplinary team meeting within the same hospital. The patient would then meet a third consultant in the specialist hospital for a discussion and be operated on by a fourth. In contrast, before the centralization, that surgeon would be present from the patient’s first to last appointment sometimes over as long as three years of treatment.

Familiarity and faith in the close working relationships between surgical and non-surgical colleagues was also affected by the centralization. One participant highlighted that it was hard to recreate this sort of community artificially:

I think when you have got your surgeons in one place and your oncologists in a different site you lose that close working relationship…You lose some of that sort of natural teaching and sort of development in the department which happens naturally. You can try and make it happen artificially by arranging meetings and things, but it’s just not the same. (oncologist, August 2018)

#### Long-term subtractive change: Loss of staff, trainees and autonomy

Staff at local sites perceived that centralization meant their services became devalued. This made continuation of routine clinical activity challenging, and there were concerns about decreasing standards of patient care. For example, local centres had to provide care for patients with postoperative complications. Due to their status as local centres, they did not have surgical staff with the right skills to manage them, and staff from the specialist centres would not come out to see the patients locally:

It’s much more complex than it ever was. The surgeons from the [specialist centres] should come out to the sites where the patients actually live rather than making the patients travel to them...Again, complications, complications come back to us. All the complications of their surgery or whatever bounce back to us, which is a shame because we haven’t got the surgeons now. We’ve been de-skilled, so the surgeons here will slowly over the years not have the skills to sort that out. (surgeon, November 2016)

#### Loss of high-calibre staff

Surgical participants at local sites perceived benign activity as less prestigious and interesting to ambitious professionals, and therefore, they were losing staff to specialist centres. Hospitals were also no longer ‘able to attract high-calibre’ staff nor able to retain them (surgeon, February 2017). Another surgeon commented:

We’re struggling to recruit new consultants because we can’t offer a sub-specialty service…Having no sub-speciality interest available to them, or very little, does not make the job attractive (surgeon, November 2018)

This effect extended to nursing staff, who were perceived as being more interested in specialist environments. As one surgeon said:

We find it very hard now to recruit CNSs [clinical nurse specialists] because we’re not really a cancer centre, so CNSs will go to places where they see centres, so they will go to [hospital]. So to recruit here for CNSs has been a nightmare. We’ve been on recruits after recruits, but, of course, why should somebody want to be a CNS here when they’re not doing all the sexy stuff? Can’t blame them, so it’s…made recruiting very difficult. (surgeon, November 2016)

Concerns were raised about the risks to patient safety, with fewer skilled staff in the local site. For example, patients being readmitted locally in an emergency with complications which required specialist surgical knowledge or skills would be at risk (surgeon, follow-up interview, April 2019). The process was perceived as gradual:

We haven’t got the surgeons now, we’ve been de-skilled so the surgeons here will slowly over the years not have the skills to sort that out…There are situations now which are more dangerous because the surgical skills are being lost. (surgeon, November 2016)

#### Loss of clinical trainees

Participants noted that the loss of specialist surgical activity also gave them less power to recruit medical trainees. Trainee positions are determined through relationships between the network and training organizations (e.g. NHS deaneries). Staff reported widespread vacancies, increased use of locum doctors and feelings of impermanence. Some hospitals were described as ‘no longer attractive for junior doctors to come in for training’ (surgeon, October 2016). This included medical trainees, who require exposure to different types of work during their training, which the local sites were unable to provide:

There isn’t much to attract a prospective consultant or a trainee to the unit unless they just want to learn very general urology work, which not many trainees want to do…Certainly, not many consultants’ ambition when they start their training is to end up in a small unit not doing any specialist work. (surgeon, follow-up interview, March 2019*)*

As well as medical trainees, participants perceived that they had lost important capacity and resources in perioperative care such as anaesthetic trainees. Furthermore, participants worried that this put their remaining activity at risk, and felt that this had caused delays for patients, lower standards of care and loss of income to the Trust. The problem was seen to be particularly problematic for surgical trainees:

It’s de-skilled us in pelvic surgery, it’s de-skilled training, our trainees don’t get exposed to pelvic surgery so they really are getting de-skilled. So we’ve got whole generations of surgeons coming through who’ve never really done any big major surgery, so that’s very poor. (surgeon, January 2017)

#### Loss of autonomy in decision-making

Surgeons who remained at local sites incurred further perceived loss of status as decision-makers around patient care. From an early planning stage, it was agreed that professionals at the specialist sites would be given responsibility for deciding on patient treatment options (‘It was not always necessary for decision-making and delivery of cancer care to be in the same place’, Pathway Board Minutes, July 2012). After implementation, this resulted in perceived loss of autonomy for local doctors:

Our local doctors, they felt like our rights have been taken over. You see, we have very experienced doctors and they decided, okay, this patient has this grade of cancer, this stage of cancer, this man needs surveillance…We still need to discuss this information with the cancer centre to double check whether my decision is right or not…It’s kind of double checking, so our doctors feel like, you know, they have taken over ruling authority. (clinical nurse specialist, May 2018)

Loss of autonomy was felt acutely as a loss of ‘rights’, with decision-making characterized as a crucial part of the professional identity of a doctor. Participants in specialist centres did not share this sentiment, and perceived the local sites as part of the multidisciplinary decision-making (e.g. surgeon, July 2016). This suggests that loss is not only an experience of changes that take place, but also related to perceived lower status as a local site.

### Emotional repercussions of subtractive change: Loss of self-image, status and motivation

#### Losing the bid: Loss of face

Loss of face is a feeling of decreased self-image, often in a situation where someone has struggled to maintain a position of responsibility.^32^ At the beginning of the process of reorganization, hospitals hoping to host a specialist surgical centre were required to submit a bid. While six bids were received, only four were chosen, and this was experienced as a loss of face by staff in those hospitals that were unsuccessful. Participants described their feelings of failure to represent and support colleagues within the Trust:

Everyone thinks you’re a failure then. You’ve failed, because you didn’t bring it home. And that goes for the team and the surgeons and the site…It really felt like you failed to deliver on something that you should have been able to get (oncologist, July 2018).

The emotions accompanying this loss may have been exacerbated by the visible nature of the bidding process to colleagues inside their own Trust and peers across the region, and the values associated with being a specialist centre.

#### Loss of status and aspiration

Losing the bid to be a specialist surgical centre also had an impact on the internal career narrative of some surgical staff, who felt that status as a ‘successful team’ had been lost in the period just after the reorganization. For instance, this surgeon suggested:

I’m sorry if I appear to be negative but… you have to appreciate that from my point of view I came here, I built something up over many years and we had very good results and very good outcomes and I had always been led to believe that if you had good results and good outcomes then you would do well. But unfortunately our outcomes have not been considered and everything that I ever built up has been taken away…and I have nothing anymore (surgeon, October 2016).

Here, the high degree of emotion appeared to be related to the loss of personal status, which threatened the staff member’s identity.^13^ This is a specific challenge with centralization, and MSC more widely, where services may experience loss *despite* performing well in their day-to-day work (here, being a surgical centre). This exerts a particular emotional impact:

So what we have done is we have taken something really good - and certainly I’m talking about this collaborative, this is not a generalisation about other collaboratives etc. etc. - we’re quite unique, and we have destroyed it. (surgeon, follow-up interview, April 2019)

This particular emotion may have become less intense as individuals adapted to the new system; indeed, some staff went on to work at the newly designated specialist centre. In their follow-up interviews, some interviewees described experiencing positive status in these new roles.

I adapted - I became a laparoscopic surgeon. I did 200, 300 laparoscopic operations. Then [i.e. previously], I was lagging behind because the world was going robotic, and this change of gear gave me the ability to be back at the forefront again. (surgeon, March 2017)

#### Loss of motivation and reward

While specialist surgery was centralized at specialist sites, other forms of surgery (e.g. benign work) continued at the local sites. One of the consequences of not being able to practise specialist surgery was the feeling of having lost the rewards of this type of work. Some nurses reported that loss of specialist surgical activity was demotivating, whereas others were not really affected by the changes: ‘We are patient advocates. So where the patient goes, for me, as long as I’m there to support them, it doesn’t really matter’ (clinical nurse specialist, March 2017). However, this nurse joined the institution when changes were already underway, suggesting that their organizational identity and site attachment had not been challenged by the centralization process.^12^ This highlights how the loss of motivation and reward individuals experience may be reliant upon their individual circumstances and their association with the institution before the changes occurred.

### Support and coping strategies offered

We identified various strategies offered during the implementation phase, usually by the central leadership team and other managerial roles. Anticipation or expression of loss from staff at local sites was often countered by an offer of instrumental support – for example, offering joint contracts, collaborative interventions or educational opportunities. Emotional support was mentioned far less often, particularly in relation to short-term loss experiences, and normally in the context of supporting other members of the implementation team.

### Coping with short-term loss: Persuasion

Central leadership figures acknowledged the emotional aspects of loss. They characterized their own role in several different ways, including ‘bridging’ and ‘persuading’. Ultimately, leaders had such a strong belief in the intended benefits of the centralization that they relied on this as a way of rationalizing the necessary emotional difficulty of the process. For example, one director suggested that people should not be judged for their emotional reactions, but balanced this against the fact that the changes were ‘wanted’:

I think you have to understand the emotion and not say people are right or wrong, but just relentlessly try and, well, be transparent…I think that we try to play that role bridging between commissioners and providers [i.e. hospitals] and between different providers and with the public to try and help through the commissioner-led new models of care that the providers, to be fair, wanted to enact (network manager, January 2016)

Leaders sometimes portrayed concerns about loss as resistance. The leaders gave support to keep people ‘on board’ through persuasion and collaborative approaches:

The biggest thing was persuading people and keeping them on board when they didn’t think it was a good plan…Making sure they’re involved in the decisions around what is the programme going to be, so that they feel that the end game is something that they have owned even though they didn’t like the idea in the first place. (senior hospital manager, April 2016)

Another participant said she drew on altruism to help promote the changes:

As much as you like to get everybody’s emotional involvement, I think sometimes it’s kind of like looking at the greater good, in terms of this is necessary…and just selling that for what it is. I think one of the hindrances that we had is because of resistance; otherwise, some of these things could have happened many years ago. (clinical nurse specialist, March 2017)

Similarly, another leadership figure felt that dwelling on loss was unhealthy for the service, and offered support through trying to get local staff to refocus on positives relating to the new service:

If you get locked into this focusing on what you feel you’ve lost, you have to acknowledge that and work through it, but if you get stuck with that, then it does tend to be, I think, it can prevent the service thriving. (clinical director, August 2016)

Therefore, acknowledgement of emotional reactions to the short-term processes of the centralization was broadly characterized in terms of overcoming resistance. Support was offered only in relation to the promise of a successful new system and in enabling the work of pathway directors who had a particularly stressful set of activities in persuading others. However, the promise of a new, effective system may have helped some staff members to forge new identities after the change.

### Coping with long-term loss: Instrumental support

From 2016 to 2018, centralization leaders provided a multidisciplinary team ‘improvement programme’ to develop common protocols for decision-making about patient pathways, but also to support clinicians in local sites. Local leaders were assigned coaches and operational support to make improvements in standardizing multidisciplinary team protocols. This may have mitigated the loss of autonomy reported in the theme of long-term subtractive change; however, we did not ask about this specifically in our interviews.

In order to bring the requisite expertise to specialist centres while mitigating the loss of staff at the local sites, joint contracts were advertised, so that some surgeons, oncologists and clinical nurse specialists were able to work in both specialist sites and their original local site employer. This meant that some team members retained familiarity with each other, and patients experienced greater continuity, through contacts with the specialist and local sites. These measures were effective in overcoming long-term loss: surgeons with joint contracts were better able to cope with professional loss if they were provided with the opportunity to gain new skills by working at the specialist centre. As a result of this, they felt like they had gained from the reorganization. For instance, one surgeon from a local site with a joint contract to a specialist site said:

I evolved. There is a process of evolution. Those who don’t go through that process of evolution stagnate and become unsuccessful…I was lagging behind because the world was going robotic and this change of gear gave me the ability to come back to the forefront again. (surgeon, March 2017)

Despite the success of this support measure, there was a perception that these contracts were not open to all. Some suitably qualified surgeons were initially offered the opportunity and then felt prevented from doing so as contracts were offered to younger surgeons:

This is a bone of contention for [our hospital], that in the other units a surgeon from that unit is going down [to the specialist centre] and doing the operation. In our unit actually that hasn’t been allowed (surgeon, May 2017)

For those who took up the opportunity to work at the specialist sites, there were also unintended consequences, with added stress for employees with joint contracts through the logistics of travelling between sites:

We had an agreed job plan, and obviously working across different sites is always difficult for anybody. So personally it’s difficult, because I’m having to go to different sites, so that often happens between sites when you’re working in both sites. (surgeon, follow-up interview, February 2019)

This highlights that resources that mitigate stress and loss at an organizational level may still induce stress at an individual level (e.g. increased workload or travel time), as staff struggle to cope in difficult circumstances.

In other cases, instrumental support measures were not delivered. For example, leaders suggested that consultants from specialist sites could hold joint posts with the local centre, but specialist surgeons were reluctant to travel to the local unit. The hospital was consequently running on a locum-based service, and the lack of permanent doctors was perceived to be a risk to the long-term stability of the hospital (manager, in first and follow-up interviews, July 2018 and February 2019). Plans were made to mitigate some professional losses by putting trainees on rotation across specialist and local hospitals, but this arrangement had not been put into place by 2019:

One of the discussions that we had was that we will have trainees. We will rotate the trainees across the two hospitals, which is again part of looking at staff training, the third education, which has not happened. And if a trainee had a rotation that included working in both the hospitals as one job, then the impact would be less. (surgeon, follow-up interview, February 2019)

This demonstrates that instrumental resources, such as training rotations, may have been more difficult to implement than other aspects of the system change. Leaders may not have prioritized these sorts of measures to mitigate the stress of losses consistently.

## Discussion

Immediately following centralization, staff experienced subtractive changes such as loss of activity, skill use and interaction with familiar team members. Over time, staff at local sites perceived shrinking, de-skilled and destabilized teams. Both individual staff members and their host organizations felt devalued, and people experienced loss of status and motivation.

Our results also highlighted how leaders put some instrumental measures in place in the centralization to mitigate these losses through joint contracting, surgical skill development opportunities and trainee rotation. However, these measures were partly undermined by feelings of inaccessibility (e.g. not all surgeons felt encouraged to apply for joint contracts) and negative individual consequences (e.g. increased workload or travel time). Relatively little emotional support was offered, and emotional reactions to the centralization were often characterized as resistance, to be overcome through persuasion and appeals to the success of the new system. Instead, leaders of large-scale change should anticipate and empathize with these feelings, and offer enhanced support of different types (i.e. instrumental, informational, emotional and understanding/validation)^17^ to help loss sites manage the change effectively. Furthermore, resources that generated a benefit to individuals (such as joint posts) could be to the detriment of the local organization, and vice versa.

Professionals reported tangible anxiety that patient experience and perioperative outcomes had worsened through the de-skilling of staff, and that the organization was severely compromised; however, specific examples of patient dangers were not given. Our study did not collect data that could substantiate or refute these anxieties. Another study of MSC noted that being able to see improvements ‘on the ground’ reduces clinicians’ fears;^33^ however, the improvements may be less visible in local sites. For context, these findings about loss come from the wider dataset, whereas most interviewees (including those in local units) felt the reorganization was positive and that conducting a higher volume of specialist surgery at a designated centre was the best option to maximize patient benefit.

This analysis builds on previous studies of major system and organizational change by reinforcing the conceptualization of change as a stressor^6,10^ and a loss.^13^ Models of leadership change, such as Bridges et al., also highlight that emotional reactions evolve over time, beginning with feelings of being threatened, expressions of grief and sadness, followed by openness to change and the establishment of new routines.^34^ We did not observe some of the features of change outlined in this model – such as feeling distracted or bargaining – which may be because participants forgot these initial experiences by the time we interviewed them. Our study makes a novel contribution by highlighting the competitive processes associated with loss incurred in centralization, restricting specialized activity to particular individuals and creating a hierarchy by giving decision-making powers and resources to specialized sites. These findings are relevant to other forms of loss, such as decommissioning.^35,36^

Our findings also concur with studies that say leaders of MSC have a responsibility to engage with their stakeholders in the emotional repercussions of change. New ways of thinking about leadership suggest that emotional connectedness and values are important,^26^ as individuals feel more resistant to MSC when it is discordant with their own values.^37,38^ Our study suggests that values are important but potentially insufficient in building the resilience needed to cope with change across the whole system, as they cannot account for abrupt changes in team composition and staffing shortages.^39^ We also echo concerns articulated by Fraser et al. about leaders’ use of clinical arguments or evidence of ‘success’ in persuading stakeholders to drive home MSC.^40^ In our own study, participants agreed with centralization in principle, but resistance was generated by concern for the long-term success of organizations and individuals.

### Implications for practice

Leaders of this MSC were able to centralize specialist cancer surgery, with the aim of improving patient outcomes. However, they inadequately managed the stress of loss, particularly in terms of providing emotional support in the implementation phase of the process. Leaders of MSC should carefully consider the timing of planned indirect changes such as training and skills development. If these are deprioritized until after the main service changes have occurred, they may never be implemented, leading to staffing gaps and risks to patient safety.

It is also the responsibility of leaders of centralized services to consider social interactions and team dynamics. More could be done at the local level to help individuals cope with loss of face and status. This could include opportunities for staff to express their feelings, obtain support from professional bodies, or engage in the co-production of practical solutions (e.g. creating a multi-site rotation plan for trainees that would support workforce deficits in local sites).^13^

### Limitations

There are several limitations with this paper. First, it is based on a focussed analysis of a larger dataset where the original research questions were broader. As such, it is likely that those wider topics might have limited some of the feedback on loss we received. For example, some participants may have had more to say about the way loss was managed by implementation leads if we had specifically asked about this.

Second, there are issues with the timing of the interviews. They were conducted after the change had been completed and, thus, were retrospective and could have been influenced by recall bias. To reduce this risk, we used documentary evidence to complement interviewees’ narration of past events. Moreover, our data were limited to a three-year window after the change had been completed. Attitudes towards loss may change further over a longer time period.

Third, despite our inclusive sampling strategy (guided by our clinical collaborators who work in the studied services), we did not recruit clinical trainees and students for their perspectives on the richness of the training environment in the local sites.

Fourth, we did not consider the emotions experienced by stakeholders who did not experience loss. Emotions are mixed in major system change, for ‘winners’ and ‘losers’ in centralization. Nevertheless, this article is specifically focussed on loss in terms of practical subtractive changes and the stress-coping mechanisms associated with these.

Fifth, our study analysed the experience of loss in relation to MSC in a specific health care area and in a predominantly urban setting. Further research could explore MSC in other specialities and contexts and look at anticipated loss in the planning stages, as well as long-term perspectives.

## Conclusions

Stress incurred by aspects of loss in system change cannot be fully prevented. But this emotional burden can be mitigated by MSC leaders paying attention to identity change and coping strategies for individual staff members. Resources to help manage feelings of loss should be delivered concurrently with other centralization changes to mitigate the risks to implementation. Leaders also need to reconsider the narrative of ‘overcoming resistance’, considering how this may be supported by providing adequate resources to mitigate stress and loss.^41^

## Supplemental Material

sj-pdf-1-hsr-10.1177_13558196221082585 – Supplemental Material for Loss associated with subtractive health service change: The case of specialist cancer centralization in England Click here for additional data file. Supplemental Material, sj-pdf-1-hsr-10.1177_13558196221082585 for Loss associated with subtractive health service change: The case of specialist cancer centralization in England by Georgia Black, Victoria Wood, Angus Ramsay, Cecilia Vindrola-Padros, Catherine Perry, Caroline Clarke, Claire Levermore, Kathy Pritchard-Jones, Axel Bex, Maxine Tran, David Shackley, John Hines, Muntzer Mughal and Naomi J Fulop in Journal of Health Services Research & Policy

## ORCID iDs

Georgia B Black https://orcid.org/0000-0003-2676-5071

Angus I G Ramsay https://orcid.org/0000-0002-4446-6916

Cecilia Vindrola-Padros https://orcid.org/0000-0001-7859-1646

Catherine Perry https://orcid.org/0000-0002-8496-6923

Caroline S Clarke https://orcid.org/0000-0002-4676-1257

Kathy Pritchard-Jones https://orcid.org/0000-0002-2384-9475

Axel Bex https://orcid.org/0000-0002-8499-2662

Maxine G B Tran https://orcid.org/0000-0002-6034-4433

David C Shackley https://orcid.org/0000-0001-8326-2556

John Hines https://orcid.org/0000-0002-5041-7694

Muntzer M Mughal https://orcid.org/0000-0002-0086-5456

Naomi J Fulop https://orcid.org/0000-0001-5306-6140

## References

[1] Fulop NJ Ramsay AI Perry C, et al. Explaining outcomes in major system change: a qualitative study of implementing centralised acute stroke services in two large metropolitan regions in England. Implementation Sci 2016; 11: 1. 10.1186/s13012-016-0445-z PMC4891887 27255558

[2] Turner S Ramsay A Perry C, et al. Lessons for major system change: centralization of stroke services in two metropolitan areas of England. J Health Serv Res Policy 2016; 21: 156–165. 26811375 10.1177/1355819615626189 PMC4904350

[3] Best A Greenhalgh T Lewis S, et al. Large‐system transformation in health care: a realist review. The Milbank Quarterly 2012; 90: 421–456. 22985277 10.1111/j.1468-0009.2012.00670.x PMC3479379

[4] Jones L Fraser A Stewart E. Exploring the neglected and hidden dimensions of large‐scale healthcare change. Sociol Health Illn 2019; 41: 1221–1235. 31099047 10.1111/1467-9566.12923

[5] Fraser A Baeza J Boaz A, et al. Biopolitics, space and hospital reconfiguration. Soc Sci Med 2019; 230: 111–121. 31009877 10.1016/j.socscimed.2019.04.011

[6] Fulop N Protopsaltis G King A, et al. Changing organisations: a study of the context and processes of mergers of health care providers in England. Soc Sci Med 2005; 60: 119–130. 15482872 10.1016/j.socscimed.2004.04.017

[7] Garside P. Are we suffering from change fatigue? BMJ Quality Safety 2004; 13: 89–90. 10.1136/qshc.2003.009159 PMC1743816 15069212

[8] Hutchinson M Vickers MH Jackson D, et al. ‘I’m gonna do what i wanna do.’ Organizational change as a legitimized vehicle for bullies. Health Care Management Review 2005; 30: 331–336. 16292010 10.1097/00004010-200510000-00007

[9] Bell E Taylor S. Beyond letting go and moving on: new perspectives on organizational death, loss and grief. Scandinavian J Management 2011; 27: 1–10.

[10] Kiefer T. Understanding the emotional experience of organizational change: Evidence from a merger. Advances Developing Human Resources 2002; 4: 39–61.

[11] Rooney D Paulsen N Callan VJ, et al. A new role for place identity in managing organizational change. Management Commun Quarterly 2010; 24: 44–73.

[12] Wiedner R Mantere S. Cutting the cord: Mutual respect, organizational autonomy, and independence in organizational separation processes. Administrative Sci Quarterly 2019; 64: 659–693.

[13] Smollan R Pio E. Organisational change, identity and coping with stress. New Zealand J Employment Relations 2017; 43: 56.

[14] Corley KG Gioia DA. Identity ambiguity and change in the wake of a corporate spin-off. Administrative Science Quarterly 2004; 49: 173–208.

[15] Conner D. Managing at the speed of change: How resilient managers succeed and prosper where others fail. New York, NY: Random House, 1993.

[16] Oreg S. Resistance to change: Developing an individual differences measure. J Applied Psychology 2003; 88: 680. 10.1037/0021-9010.88.4.680 12940408

[17] House J. Work stress, and social support. Reading, MA: Addison-Wesley Pub Co, 1981.

[18] Clarke C Hope-Hailey V Kelliher C. Being real or really being someone else? Change, managers and emotion work. European Management J 2007; 25: 92–103.

[19] Vindrola-Padros C Ramsay AI Perry C, et al. Implementing major system change in specialist cancer surgery: The role of provider networks. J Health Serv Res Policy 2021; 26: 4–11. 32508182 10.1177/1355819620926553 PMC7734603

[20] Gooiker GA van Gijn W Wouters MW, et al. Systematic review and meta‐analysis of the volume–outcome relationship in pancreatic surgery. British J Surg 2011; 98: 485–494. 10.1002/bjs.7413 21500187

[21] Department of Health. Guidance on Commissioning Cancer Services: Improving Outcomes in Upper Gastro-intestinal Cancers: the Manual. London, UK: Department of Health, 2001.

[22] NICE. Improving outcomes in urological cancers: Cancer service guideline [CSG2]. London, UK: NICE, 2002.

[23] NHS England. Five year forward view. England, UK: NHS England. www.england.nhs.uk/wp-content/uploads/2014/10/5yfv-web.pdf (2014, accessed 10th January 2020).

[24] NHS Commissioning support for London. Cancer Services Case for Change. UK: NHS England, 2010.

[25] Fulop NJ Ramsay AI Vindrola-Padros C, et al. Reorganising specialist cancer surgery for the twenty-first century: a mixed methods evaluation (RESPECT-21). Implementation Sci 2016; 11: 155. 10.1186/s13012-016-0520-5 PMC5123291 27884193

[26] Bevan H Fairman S. The new era of thinking and practice in change and transformation: a call to action for leaders of health and care. England, UK: United Kingdom NHS Improving Quality LeedsUK Government White Paper, 2014.

[27] Marshall MN. The key informant technique. Family Practice 1996; 13: 92–97. 8671109 10.1093/fampra/13.1.92

[28] Fereday J Muir-Cochrane E. Demonstrating rigor using thematic analysis: A hybrid approach of inductive and deductive coding and theme development. Int Journal Qualitative Methods 2006; 5: 80–92.

[29] Gale NK Heath G Cameron E, et al. Using the framework method for the analysis of qualitative data in multi-disciplinary health research. BMC Medical Research Methodology 2013; 13: 117. 24047204 10.1186/1471-2288-13-117 PMC3848812

[30] Smollan R Pio E. Identity and Stressful Organizational Change: A Qualitative Study. In: Emotions Network (Emonet). Berlin, Germany: European Group for Organizational Studies (EGOS); 2016.

[31] Lazarus R Folkman S. Stress, appraisal, and coping. New York, NY: Springer Pub Co, 1984.

[32] Goffman E. On face-work: an analysis of ritual elements in social interaction. Psychiatry 1955; 18: 213–231. 13254953 10.1080/00332747.1955.11023008

[33] Fulop NJ Ramsay AI Hunter RM, et al. Factors influencing the sustainability of changes in London. In: Evaluation of reconfigurations of acute stroke services in different regions of England and lessons for implementation: a mixed-methods study. England, UK: NIHR Journals Library; 2019. 30789689

[34] Bridges W Mitchell S. Leading transition: a new model for change. Leader to Leader 2000; 16: 30–36.

[35] Harlock J Williams I Robert G, et al. Doing more with less in health care: findings from a multi-method study of decommissioning in the english national health service. J Social Policy 2017; 47: 543–564.

[36] Williams I Harlock J Robert G, et al. Is the end in sight? A study of how and why services are decommissioned in the English National Health Service. Sociol Health Illn 2021; 43: 441–458. 33636017 10.1111/1467-9566.13234

[37] Martin GP Currie G Finn R. Leadership, service reform, and public-service networks: the case of cancer-genetics pilots in the English NHS. J Public Administration Research Theory 2008; 19: 769–794.

[38] Hakak LT. Strategies for the resolution of identity ambiguity following situations of subtractive change. J Applied Behavioral Sci 2015; 51: 129–144.

[39] Rickles D Hawe P Shiell A. A simple guide to chaos and complexity. J Epidemiology Community Health 2007; 61: 933–937. 10.1136/jech.2006.054254 PMC2465602 17933949

[40] Fraser A Baeza JI Boaz A. ‘Holding the line’: a qualitative study of the role of evidence in early phase decision-making in the reconfiguration of stroke services in London. Health Research Policy Systems 2017; 15: 45. 28599658 10.1186/s12961-017-0207-7 PMC5466773

[41] Holmes BJ Best A Davies H, et al. Mobilising knowledge in complex health systems: a call to action. Evid Policy: A J Res Debate Practice 2017; 13: 539–560.

