# Supplementary material for: Loss associated with subtractive health service change: The case of specialist cancer centralization in England
Source: J Health Serv Res Policy. 2022 Apr 26;27(4):301–12. doi: 10.1177/13558196221082585 (PMC9548928; doi:10.1177/13558196221082585)
Supplement: sj-pdf-1-hsr-10.1177_13558196221082585 – Supplemental Material for Loss associated with subtractive health service change: The case of specialist cancer centralization in England [file sj-pdf-1-hsr-10.1177_13558196221082585.pdf]

Online Supplement 1

Table S1. Changes to local and specialist surgical sites during the centralisation

	Sites that never provided specialist surgical activity (local)	Sites that lost specialist surgical activity (local)	Sites that were selected as specialist centres (specialist)
**Prostate/bladder**	8	3	1
**Renal**	2	8	1
**Oesophago-gastric**	9	1	1+1[a]

[a]A single site was recommended for specialist OG surgery; however, a further site continued to provide OG surgery with the expectation that it would be consolidated over time. This has not yet happened at the time of publication.

Table S2. Standards and resources expected of a local and specialist centre (populated through discussions with clinical collaborators)

	**Local units**	**Specialist centres**
**Leadership**		Named leader who takes responsibility for system-wide collaborative working, and manages relationship to local units.
**Multidisciplinary team composition**	Local MDT[a] with membership as per peer review measures, e.g.:<br>• Clinical nurse specialist<br>• Surgeon<br>• Oncologist<br>• Palliative care representative<br>• MDT coordinator<br>• Radiologist<br>• Histopathologist<br>• Relevant disciplines e.g. dietician, clinical psychology.	Specialist MDT with additional members to local MDT including:<br>• Specialist surgeons<br>• Specialist oncologists<br>• Other specialists e.g. Specialist Allied Health Professionals, interventional radiologists providing advanced tumour staging techniques and non-surgical treatment options<br>• Clinical trials nurses
**Diagnostics available**	• Computed tomography<br>• Magnetic resonance imaging<br>• Ultrasound	• Nuclear medicine<br>• Positron emission tomography techniques<br>• Image-guided biopsy techniques
**Treatment options available**	• Radiotherapy (where available)<br>• Chemotherapy (if provided)<br>• Cancer-specific therapies e.g. palliative endoscopy<br>• Benign surgery<br>• Follow-up care	• Radiotherapy<br>• Chemotherapy<br>• Cancer-specific therapies e.g. palliative endoscopy, hormone therapy, non-surgical tumour ablation (e.g. cryotherapy, radiofrequency ablation, HIFU etc)<br>• Full spectrum of surgical procedures including minimally invasive surgical techniques and complex benign cases<br>• Full spectrum of intervention radiology including ablation and embolisation
**Specialist workforce**	• Dedicated oncology clinical nurse specialist<br>• Chemotherapy pharmacist	• Specialist anaesthetists<br>• Surgical outreach from the centre for post op follow up
**Education and training**	• Training for all professional members in specialist content	• Training for all professional members in specialist content<br>• Offers simulation training in new surgical techniques<br>• Conducts training in delivery of systemic therapy
**Trials and research**	• Carry out prospective audit of service and publish transparent outcomes data<br>• Participation in network audit programme and National Audits<br>• Access to clinical trials and research nursing	• Take full part in all relevant clinical trials<br>• Ensure all patients asked at first appointment if they would consider being entered into a clinical trial.<br>• Entry into trials offered at first decision to treat appointment – after histology and CT is available<br>• Carry out prospective audit of service and publish transparent outcomes data<br>• Tissue biobanking

[a]MDT = multidisciplinary team
